# The Impact of Diagnosis on Job Retention: A Danish Registry-Based Cohort Study

**DOI:** 10.1155/2015/795980

**Published:** 2015-12-01

**Authors:** Rasmus Espersen, Vibeke Jensen, Martin Berg Johansen, Kirsten Fonager

**Affiliations:** ^1^Department of Social Medicine, Aalborg University Hospital, 9100 Aalborg, Denmark; ^2^Department of Clinical Medicine, Aalborg University, 9000 Aalborg, Denmark; ^3^Department of Health Science and Technology, Faculty of Medicine, Aalborg University, 9920 Aalborg, Denmark

## Abstract

*Background*. In 1998, Denmark introduced the flex job scheme to ensure employment of people with a permanent reduced work capacity. This study investigated the association between select diagnoses and the risk of disability pension among persons eligible for the scheme.* Methods*. Using the national DREAM database we identified all persons eligible for the flex job scheme from 2001 to 2008. This information piece was linked to the hospital discharge registry. Selected participants were followed for 5 years.* Results*. From the 72,629 persons identified, our study included 329 patients with rheumatoid arthritis, 10,120 patients with spine disorders, 2179 patients with ischemic heart disease, and 1765 patients with functional disorders. A reduced risk of disability pension was found in the group with rheumatoid arthritis (hazard ratio = 0.69 (0.53–0.90)) compared to the group with spine disorders. No differences were found when comparing ischemic heart disease and functional disorders. Employment during the first 3 months of the flex job scheme increased the degree of employment for all groups.* Conclusion*. Differences in the risk of disability pension were identified only in patients with rheumatoid arthritis. This study demonstrates the importance of obtaining employment immediately after allocation to the flex job scheme, regardless of diagnosis.

## 1. Introduction

As the population ages, a growing number of elderly persons have retired from the labour market. With a large number of people aging out of workforce, it is important to keep people of working age in the labour market for as long as possible [1]. In spite of improved health and increased life expectancy, most populations have seen an increase in disability benefit [1]. The relation between sickness absence and labor market exclusion is a complex issue, informed by a complicated interaction of individual circumstances (e.g., gender and age), working conditions, and societal conditions [2–4]. Labour market characteristics such as unemployment and labour market flexibility may influence the rate of sickness absence [5] and, for the individual, long-term sickness absence has an impact on risk of future disability pension and mortality [2, 6]. Helping sick-listed persons back into employment is important not only for the community, but also for maintaining work based identity and social networks [7].

The flex job scheme was introduced in Denmark in 1998 to keep people of working age with a permanent reduced work capacity employed rather than transitioning them to permanent, passive recipients of a disability pension. Persons eligible for the flex job scheme experienced a permanent reduction in working capacity by at least 50% and have previously exhausted all other avenues of obtaining ordinary employment. Employers hiring workers who have been approved for the flex job scheme are entitled to a wage subsidy equivalent to either half or two-thirds of the agreed upon wage. The number of persons declared eligible for the flex job scheme has increased over the years, with no notable decline in persons awarded a disability pension [8, 9].

When declared eligible for the flex job scheme, a person can either be employed as a flex job holder or be unemployed and receiving unemployment benefits. A recent study found that one-third of persons declared eligible for the flex job scheme were unemployed for more than 3 months; 23% did not obtain employment before they were allocated a disability pension [9]. The reasons for the reduced working capacity may be important for job retention; the impact on job retention may differ for different health problems. Previous studies have shown that rheumatoid arthritis patients must be employed to benefit from vocational rehabilitation programs [10–13]. For persons with low back pain, age and long-term sick leave are risk factors for not returning to work after joining a rehabilitation program [14].

The intentions of the flex job scheme were good since more people were to stay at the labour market despite reduced working capacity. However, patients with some diagnoses may benefit more from the flex job scheme than others. For example, as compared to a localized disability, a generalized disease may have a greater impact on job retention due to the need for more widespread changes at the workplace. A potential life-threatening disease might have impact on job retention. Ischemic heart disease is a potentially life-threatening disease, which can lead to, for example, depression resulting in a negative impact on job retention [15]. From clinical work, it is the impression that job retention in the flex job scheme for individuals with functional disorders is comparable to those with a potentially life-threatening disease. Functional disorders include a variety of bodily symptoms recently introduced as bodily distress syndrome [16]. The potential mechanism in bodily distress syndrome involves pathophysiological, psychological, and social components, necessitating a biopsychosocial approach to treatment.

In the present research, we studied the association between select diagnoses and the risk of disability pension among persons who are eligible for the flex job scheme to evaluate the impact of disease localization (rheumatoid arthritis versus spine disorder). Additionally, we investigated the difference in job retention between individuals with a well-defined and potentially life-threatening disorder (ischemic heart disease) and those with an unspecific disease with physical symptoms, not explained by well-recognized medical illness (functional disorder).

## 2. Methods

### 2.1. Study Population

The study population included all flex job scheme eligible adult persons residing in Denmark between January 1, 2001, and March 31, 2008. The study population was followed from inclusion until disability pension or any of the following events: flex benefit (comparable to early retirement benefit; Danish: Efterløn), old age pension, emigration, death, or end of follow-up (five years after being declared eligible for the flex job scheme). The study population was identified using the Ministry of Employment's DREAM database, which contains data on all recipients of social benefits in Denmark since 1991. Used in several prior public health research studies, the database is updated weekly and includes specifics on the kind of benefits received [17, 18]. This study focused on the codes for flex job (both flex job holders) (codes 771–773, 779) and unemployment benefit recipients (codes 740–749), flex benefit (code 796), disability pension (codes 793, 761-762, and 769), and old age pension (code 998) (DREAM version 10, 2006).

The Danish National Health Service provides tax-funded healthcare and social welfare for all citizens. Using the unique civil registration (CPR) number, data were linked to the Danish National Patient Registry (NPR), which contains data on all hospital admissions since 1977 and all outpatient visits since 1995. Data were extracted on diagnoses (ICD-10) during the 3 years prior to being declared eligible for the flex job scheme. The choice of a three-year period was informed by the long period often used for prescreening, treatment and needs assessment before a person is determined to be eligible for the flex job scheme. Data were linked to social and demographic data obtained from the Integrated Labour Market Research Database (IDA), Statistics Denmark. For each individual, baseline data were extracted from the IDA for the calendar year before the individual was considered eligible for the flex job scheme.

**Figure 1:**
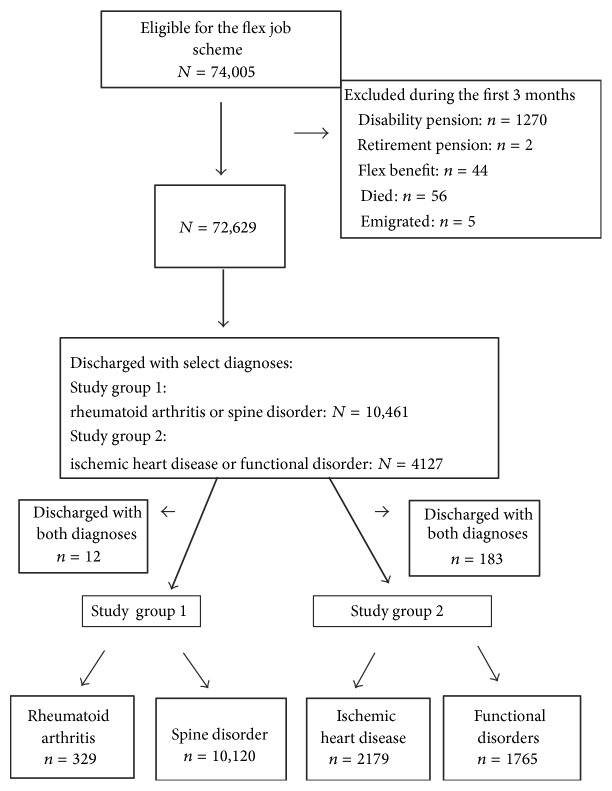
Selection of study participants from adults enrolled in the flex job scheme in Denmark from 2001 to 2008.

We identified the following groups of patients from the NPR: rheumatoid arthritis (RA) M05, spine disorder (SD) M40–54, ischemic heart disease (IHD) I20–25, and functional disorder (FD) S13.4, O26.7, M79.0, and G93.3. In order to determine if disease localization impacts job retention in the flex job scheme, we compared patients with RA and SD. Patients with both diagnoses were excluded. In order to determine whether having a potentially life-threatening disease differed from having an unspecific disease with respect to job retention in the flex job scheme, we compared patients with IHD and FDs. Patients with both diagnoses were excluded (Figure 1).

### 2.2. Covariates

Information about covariates was obtained from the IDA, including data on gender, age, education (based on HFFSP, highest educational level: primary school (main code 10)), supplementary primary education (main codes 20, 25), short-term education (main codes 35, 40), medium-term education (main codes 50, 60), or long-term education (main codes 65, 70), marital status (single or cohabiting/married), and home-dwelling children. Information on ethnic background (Danish or non-Danish) and region of residence (the five administrative regions of Denmark: Northern Jutland, Central Jutland, Southern Denmark, Zealand, or Capital) was obtained from the DREAM database.

Participants were categorized as either flex job holders or unemployment benefit recipients based on data from the 3 months after being declared eligible for the flex job scheme. The flex job holders were defined as persons who were employed in a flex job within the first 3 months after being declared eligible for the scheme. The unemployment benefit recipients were defined as persons who received unemployment benefits throughout the same time period. The starting point of follow-up was 3 months after an individual was declared eligible for the flex job scheme.

### 2.3. Employment Time Ratio

In order to study the influence of diagnosis on the degree of employment before disability pension, we calculated the* employment time ratio* in each diagnosis group as follows: each individual contributed with a number of weeks employed from 3 months after being declared eligible for the flex job scheme (employment time). The sum of all employment time in each diagnostic group was divided by the total observation time to estimate the group-level proportion of weeks employed in the flex job scheme.

### 2.4. Statistical Analysis

The primary endpoint was disability pension. The influence of diagnosis was assessed in a Cox regression model. SD was chosen as the reference when comparing RA to SD and FD was chosen as a reference when comparing IHD to FD. Crude hazard ratios (HRs) were estimated, as well as HRs adjusted for covariates. Each estimate was reported with the corresponding 95% confidence interval (CI). Due to violations of the proportional hazards assumptions for some nonexposure covariates, nonproportional associations were allowed for the relevant covariates by letting the effect of these variables vary during follow-up. Next, the HRs were stratified for employment status (flex job holder or unemployment benefit recipient) in the 3-month period after being found eligible for the flex job scheme. Crude and adjusted HRs were estimated along with the corresponding 95% CIs.

Employment time ratios were reported along with the corresponding 95% CI, which was calculated using the bootstrapping method [19]. In addition, the employment time ratios were stratified for employment status after 3 months (flex job holder or unemployment benefit recipient).

Statistical tests were two-sided, and *P* < 0.05 was considered significant. Statistical analyses were performed using Stata version 13.

## 3. Results

A total of 74,005 persons were declared eligible for the flex job scheme during the study period. During the first 3 months, 1376 persons were excluded from the study due to disability pension, retirement pension, flex benefit, emigration, and death. From the remaining 72,629 persons, two study groups were identified (Figure 1).

The baseline characteristics of the study populations are provided in Table 1. Patients with RA were more often women, had higher education, and were more often employed within the first 3 months after being found eligible for the flex job scheme compared to patients with SD. Patients with IHD were more often men, older, and more often employed within the first 3 months after being found eligible for the flex job scheme compared to the patients with FDs.

### 3.1. Risk of Disability Pension

Among patients with RA, 17% were allocated disability pension compared to 23% of patients with SD. In the adjusted model, there was a lower risk of being allocated disability pension (HR 0.69) if discharged with RA compared to SD (Table 2). When stratified for employment status in the first 3 months, we found no significant difference between RA and SD for the flex job holders or unemployment benefit recipients, though there tended to be a lower risk of being allocated disability pension among flex job holders with RA.

Among patients with IHD, 24% were allocated disability pension compared to 22% of patients with FDs. In the unadjusted model, patients with IHD seemed to be at a higher risk of being allocated disability pension than patients with FDs, but the association disappeared in the adjusted model (HR 0.96). This was also seen when stratified for employment status at baseline.

### 3.2. Time Employed before Disability Pension

Overall, patients with RA were employed significantly longer before disability pension than the other study groups (Table 3). The flex job holders with RA had significantly more weeks of employment (employment time ratio 0.91) than flex job holders with SD (employment time ratio 0.86). No significant difference was found for the unemployment benefit recipients.

For flex job holders with IHD and FDs, there was no significant difference in employment time. Unemployment benefit recipients with IHD were employed for significantly fewer weeks (employment time ratio 0.40) than unemployment benefit recipients with FD (employment time ratio 0.46).

For all study groups, the employment time ratio was significantly different when comparing flex job holders to unemployment benefit recipients, with the highest employment time ratio among flex job holders.

## 4. Discussion

Our study identified a reduced risk of disability pension in patients with RA as compared to those with SD. In contrast, there were no observed differences when comparing IHD to FDs. When stratifying for employment status, no significant differences were found, although there was a tendency towards a reduced risk among patients with RA. Moreover, patients with RA were employed longer than those with other diagnoses, exhibiting the highest degree of employment among flex job holders.

To the best of our knowledge, this study is the first to evaluate the impact of diagnosis on the flex job scheme. The use of registry-based, systematically collected data allowed us to include every eligible adult in the country and improved follow-up, minimizing the risk of selection bias. This technique also comes with limitations. Discharge diagnoses can vary in quality, likely with the highest validity in the most specific diagnosis group (i.e., RA). Additionally, we received no data on patients treated outside of the hospitals. Patients with RA may have more contact with hospitals than those with SD, IHD, or FDs. For these diagnoses, we likely only have information on the most severe cases, which may explain the differences between the groups. Finally, we have no confirmation that the discharge diagnosis is the reason for eligibility for the flex job scheme. We extracted the primary diagnosis for the discharge; therefore, we only included the main health problem resulting in contact with the hospital for that particular admission, excluding other health factors that may have played a role in eligibility. Patients in our study may have had other admissions or contact with general practitioners about important health problems in the 3 years prior to inclusion. The estimates were adjusted for covariates, but we cannot rule out the influence of other covariates, such as financial compensation and comorbidities, including psychosocial problems.

Our findings indicate an association between different diagnoses in the locomotor system and job retention. Having RA compared to SD reduced the risk of being allocated disability pension. When stratifying for employment status it was demonstrated that the reduced risk was most pronounced among persons who were employed 3 months after being declared eligible for the flex job scheme. Other studies have shown that persons with a rheumatic disease must be employed to obtain benefits from vocational rehabilitation [10–13].

We found no significant differences between IHD and FD, except for the number of weeks employed before disability pension for the unemployment benefit recipients. IHD is a potentially life-threatening disease, which can lead to posttraumatic stress disorder- (PTSD-) like symptoms, with depression or symptoms of depression more prevalent among survivors than in the general population [15]. This may have a negative impact on job retention, employment status, and productivity [20, 21]. Our study suggests that patients with FDs manage equally well regarding the risk of disability pension compared to patients with IHD, even when including covariates in the model. FDs are characterized by the need for a biopsychosocial approach to treatment including cognitive behavioral therapy and graded exercises [22], which may require more intense individually targeted support and follow-up. To improve differentiated rehabilitation, we need a greater understanding of the factors important for job retention for persons with reduced work capacity in the labour market.

In conclusion, our findings revealed only minor differences in the risk of disability pension across select diagnoses, with the exception of patients with RA. The study further demonstrated the importance of obtaining employment immediately following allocation to the flex job scheme, regardless of diagnosis. If the flex job scheme is to be an effective tool for keeping people with reduced work capacity employed, it should focus on assigning eligible individuals to new employment opportunities soon after joining the program.

## Figures and Tables

**Table 1:** Baseline characteristics of study participants.

Diagnosis	Study group 1		Study group 2		Total
	Rheumatoid arthritis	Spine disorders	Ischemic heart disease	Functional disorders	
	*N* = 329	*N* = 10,120	*N* = 2179	*N* = 1765	*N* = 72,629
	*n* (%)	*n* (%)	*n* (%)	*n* (%)	*n* (%)
Gender					
Female	251 (76)	5887 (58)	717 (33)	1327 (75)	44,182 (61)
Male	78 (24)	4233 (42)	1462 (67)	438 (25)	28,447 (39)
*Age at t0, mean (SD)*	*47.7 (8)*	*46.4 (8)*	*52.7 (6)*	*41.2 (9)*	*46.4 (10)*
Education					
Primary school	99 (30)	3491 (35)	732 (34)	569 (33)	26,212 (37)
Supplementary primary school	12 (4)	248 (3)	54 (3)	80 (5)	2490 (4)
Short-term education	155 (47)	4984 (50)	1054 (50)	853 (49)	31,911 (45)
Medium-term education	55 (17)	1046 (11)	241 (11)	205 (12)	9031 (13)
Long-term education	6 (2)	125 (1)	45 (2)	26 (2)	1470 (2)
Marital status					
Single	83 (26)	2497 (25)	532 (25)	485 (28)	22,818 (33)
Cohabitating/married	242 (75)	7410 (75)	1602 (75)	1245 (72)	47,142 (67)
Children living at home					
Home-dwelling children	140 (43)	4515 (46)	551 (26)	962 (56)	27,290 (39)
No home-dwelling children	185 (57)	5392 (54)	1583 (74)	768 (44)	42,670 (61)
Region					
Capital	72 (22)	1862 (18)	514 (24)	403 (23)	14,447 (20)
Zealand	67 (20)	1331 (13)	341 (16)	319 (18)	10,872 (15)
Southern Denmark	57 (17)	2865 (28)	529 (24)	303 (17)	18,730 (26)
Central Jutland	97 (30)	2587 (26)	564 (26)	497 (28)	19,989 (28)
Northern Jutland	36 (11)	1475 (15)	230 (11)	243 (14)	8582 (12)
Ethnic background					
Danish	314 (95)	9285 (92)	2000 (92)	1637 (93)	67,123 (92)
Non-Danish	15 (5)	835 (8)	179 (8)	128 (7)	5506 (8)
Employment status					
Flex job holders	239 (73)	6303 (62)	1559 (72)	1085 (62)	48,151 (66)
Unemployment benefit recipients	90 (27)	3817 (38)	620 (29)	680 (39)	24,478 (34)

**Table 2:** Risk of disability pension for different diagnoses stratified by employment status at baseline.

	*n* (%)	Crude HR (95% CI)	Adjusted^*∗*^ HR (95% CI)
*Overall*			
Study group 1			
Rheumatoid arthritis	55 (17)	0.70 (0.54–0.92)	0.69 (0.53–0.90)
Spine disorders	2258 (23)	1 [ref.]	1 [ref.]
Study group 2			
Ischemic heart disease	490 (24)	1.19 (1.04–1.36)	0.96 (0.84–1.10)
Functional disorders	381 (22)	1 [ref.]	1 [ref.]
*Status after 3 months: flex job holders*			
Study group 1			
Rheumatoid arthritis	26 (11)	0.69 (0.47–1.02)	0.69 (0.47–1.02)
Spine disorders	956 (16)	1 [ref.]	1 [ref.]
Study group 2			
Ischemic heart disease	253 (17)	1.31 (1.08–1.60)	1.03 (0.84–1.27)
Functional disorders	155 (15)	1 [ref.]	1 [ref.]
*Status after 3 months: unemployment benefit recipients*			
Study group 1			
Rheumatoid arthritis	329 (34)	0.90 (0.62–1.29)	0.88 (0.61–1.27)
Spine disorders	1302 (36)	1 [ref.]	1 [ref.]
Study group 2			
Ischemic heart disease	237 (41)	1.42 (1.18–1.70)	1.02 (0.85–1.23)
Functional disorders	226 (35)	1 [ref.]	1 [ref.]

^*∗*^Adjusted for gender, age, education, marital status, home-dwelling children, ethnic background, and region of residence.

**Table 3:** Degree of employment (*employment time ratio* ^*∗*^) in the flex job scheme before disability pension for different diagnoses stratified for employment status at baseline.

	Total	Study group 1		Study group 2	
		Rheumatoid arthritis	Spine disorders	Ischemic heart disease	Functional disorders
	*N* = 72,629 Proportion (95% CI)	*N* = 329 Proportion (95% CI)	*N* = 10,120 Proportion (95% CI)	*N* = 2179 Proportion (95% CI)	*N* = 1765 Proportion (95% CI)
Overall	0.74 [0.74–0.74]	0.82 [0.79–0.85]	0.74 [0.74–0.75]	0.76 [0.75–0.78]	0.73 [0.71–0.74]
Flex job holders	0.84 [0.84–0.85]	0.91 [0.89–0.94]	0.86 [0.85–0.86]	0.87 [0.86–0.88]	0.86 [0.84–0.87]
Unemployment benefit recipients	0.47 [0.46–0.47]	0.51 [0.43–0.59]	0.49 [0.48–0.50]	0.40 [0.36–0.43]	0.46 [0.43–0.49]

^*∗*^Employment time ratio: number of weeks in the flex job scheme for each diagnostic group divided by the total observation time.
